# Supplementary material for: The Role of SHIP1 on Apoptosis and Autophagy in the Adipose Tissue of Obese Mice
Source: Int J Mol Sci. 2020 Sep 30;21(19):7225. doi: 10.3390/ijms21197225 (PMC7582772; doi:10.3390/ijms21197225)
Supplement: Supplementary file 1 [file ijms-21-07225-s001.pdf]

The Role of SHIP1 on Apoptosis and Autophagy in the Adipose Tissue of Obese Mice

Jae Hun Jeong, Hye Min Jang, Eun Bee Choi, Yu Jeong Ahn, Hyeong Seok An, Jong Youl Lee, Kyung-Ah Park, Eun Ae Jeong, Hyun Joo Shin, Jaewoong Lee, Kyung Eun Kim and Gu Seob Roh *

Department of Anatomy and Convergence Medical Science, Bio Antiaging Medical Research Center, Institute of Health Sciences, College of Medicine, Gyeongsang National University, Jinju, Gyeongnam, Republic of Korea

Supplementary Table 1. List of common differentially expressed genes related to apoptosis and inflammation in the adipose tissue of control, diet-induced obesity, and *ob*/*ob* mice.

ID	Symbol	logFC	*p* Value
ENSMUSG00000055024	*Fga*	−6.46132	0.003641
ENSMUSG00000019843	*Fgb*	−6.12832	0.001492
ENSMUSG00000025958	*Ctla4*	−6.00097	0.000819
ENSMUSG00000020605	*Adipoq*	−3.27777	1.24E−09
ENSMUSG00000027598	*Ddit4*	−2.82899	9.91E−10
ENSMUSG00000028029	*Fgfr3*	−2.67056	1.70E−06
ENSMUSG00000022200	*Edn1*	−2.38541	6.85E−07
ENSMUSG00000034218	*Hcar2*	−2.38072	6.89E−06
ENSMUSG00000027951	*C6*	−2.32903	2.04E−05
ENSMUSG00000022508	*Gsn*	−2.0003	6.66E−05
ENSMUSG00000025809	*Gpam*	−1.99022	8.55E−08
ENSMUSG00000031511	*Cd59b*	−1.9811	7.69E−05
ENSMUSG00000006699	*Irs2*	−1.93308	2.72E−09
ENSMUSG00000027944	*Jag2*	−1.90104	0.000102
ENSMUSG00000020053	*Bnip3*	−1.88001	7.66E−06
ENSMUSG00000054717	*Dab2ip*	−1.84258	1.75E−09
ENSMUSG00000028191	*Esr1*	−1.74076	1.50E−07
ENSMUSG00000000787	*Dll1*	−1.71297	0.002202
ENSMUSG00000037405	*Egr1*	−1.70343	0.002635
ENSMUSG00000015656	*Cav1*	−1.65249	0.000548
ENSMUSG00000038160	*Cnr1*	−1.39357	0.002675
ENSMUSG00000001729	*Hoxa5*	−1.28892	0.004514
ENSMUSG00000033538	*Itga1*	−1.256	0.004139
ENSMUSG00000057789	*Fgf10*	−1.13288	0.001435
ENSMUSG00000060216	*Cd38*	−1.1312	8.22E−06
ENSMUSG00000052085	*Gpld1*	−1.12901	0.000499
ENSMUSG00000032359	*Epha2*	−1.11901	0.008496
ENSMUSG00000045005	*Irak2*	−1.10552	9.96E−05

ENSMUSG00000031628	*Erbb2*	−1.09027	0.002484
ENSMUSG00000035992	*Bmp6*	−1.03176	0.001187
ENSMUSG00000030528	*Fas*	−0.89236	0.000721
ENSMUSG00000003873	*Bcl2*	−0.70785	0.000307
ENSMUSG00000029657	*Gfer*	−0.68725	0.001407
ENSMUSG00000024659	*Birc2*	−0.58819	0.006624
ENSMUSG00000042349	*Bcap31*	−0.53533	0.008235
ENSMUSG00000024621	*Glo1*	−0.42479	0.004091
ENSMUSG00000044147	*Dnaja3*	−0.4183	0.008946
ENSMUSG00000044303	*Ep300*	0.499157	0.006547
ENSMUSG00000031616	*Fyn*	0.518861	0.004211
ENSMUSG00000068227	*Creb1*	0.549847	0.000749
ENSMUSG00000017652	*Hs1bp3*	0.560734	0.001791
ENSMUSG00000052593	*Gpi1*	0.566032	0.004435
ENSMUSG00000026596	*Itch*	0.573925	0.001668
ENSMUSG00000030707	*Aimp1*	0.579709	0.007314
ENSMUSG00000027087	*Golph3*	0.594232	0.001149
ENSMUSG00000099974	*Atm*	0.600186	0.001181
ENSMUSG00000054428	*Adar*	0.616173	0.001732
ENSMUSG00000025001	*Bcl6*	0.635089	0.003493
ENSMUSG00000024397	*Itgb1*	0.684814	0.000771
ENSMUSG00000039145	*Arhgef7*	0.711919	1.82E−05
ENSMUSG00000033792	*Cdc42*	0.736628	0.000122
ENSMUSG00000031778	*Hax1*	0.761821	0.000796
ENSMUSG00000027381	*Igf1*	0.763898	0.001868
ENSMUSG00000026928	*Hmgb2*	0.764097	0.002502
ENSMUSG00000037860	*Bcl10*	0.771833	3.31E−05
ENSMUSG00000026288	*Ddx3x*	0.787552	8.88E−05
ENSMUSG00000035042	*Icam1*	0.836098	0.003439
ENSMUSG00000032093	*Hspa8*	0.843284	0.000386
ENSMUSG00000017697	*Atg5*	0.916843	0.000266
ENSMUSG00000029771	*Akt1*	0.958977	3.15E−05
ENSMUSG00000003812	*Casp4*	0.992875	0.003007
ENSMUSG00000022831	*Bak1*	1.027466	8.87E−08
ENSMUSG00000030641	*Arrb2*	1.047866	1.25E−05
ENSMUSG00000049130	*Dock8*	1.061942	0.000124
ENSMUSG00000058715	*Ctsh*	1.089908	0.000124

ENSMUSG00000079227	*Fzd5*	1.188341	2.20E−05
ENSMUSG00000031264	*Casp3*	1.194145	2.03E−10
ENSMUSG00000025888	*Fnip1*	1.215688	1.31E−08
ENSMUSG00000029915	*Blm*	1.225639	0.00104
ENSMUSG00000036526	*Bax*	1.252833	5.59E−06
ENSMUSG00000005087	*Hsph1*	1.263258	2.48E−08
ENSMUSG00000003283	*Anxa1*	1.339567	2.61E−07
ENSMUSG00000004296	*Ikbke*	1.347374	7.56E−08
ENSMUSG00000000982	*Csf1r*	1.368415	4.32E−10
ENSMUSG00000015854	*Arf6*	1.41026	2.45E−13
ENSMUSG00000015437	*Cdkn2a*	1.425399	0.003655
ENSMUSG00000005413	*Ednra*	1.439549	9.79E−05
ENSMUSG00000032076	*Il2rb*	1.4828	0.000935
ENSMUSG00000026981	*Cd40*	1.493801	2.98E−05
ENSMUSG00000025473	*Adam17*	1.544802	4.41E−13
ENSMUSG00000026596	*Abl2*	1.564504	4.67E−09
ENSMUSG00000030707	*Coro1a*	1.567917	3.66E−12
ENSMUSG00000027087	*Itgav*	1.605422	3.06E−18
ENSMUSG00000099974	*Bcl2a1d*	1.669281	0.00017
ENSMUSG00000054428	*Atpif1*	1.673306	1.39E−08
ENSMUSG00000025001	*Hells*	1.683084	2.34E−06
ENSMUSG00000024397	*Aif1*	1.771951	5.58E−08
ENSMUSG00000039145	*Camk1d*	1.798252	1.23E−16
ENSMUSG00000033792	*Atp7a*	1.835452	1.87E−08
ENSMUSG00000031778	*Cx3cl1*	1.867246	0.001911
ENSMUSG00000027381	*Bcl2l11*	1.899143	7.95E−17
ENSMUSG00000026928	*Card9*	1.903507	1.80E−10
ENSMUSG00000037860	*Aim2*	1.966615	4.03E−10
ENSMUSG00000026288	*Inpp5d*	1.979603	8.96E−14
ENSMUSG00000035042	*Ccl5*	2.072736	1.19E−05
ENSMUSG00000032093	*Cd3e*	2.086772	0.000826
ENSMUSG00000017697	*Ada*	2.088379	2.41E−09
ENSMUSG00000029771	*Irf5*	2.132703	4.73E−15
ENSMUSG00000003812	*Dnase2a*	2.262342	3.99E−19
ENSMUSG00000022831	*Hcls1*	2.36304	1.30E−26
ENSMUSG00000030641	*Ddias*	2.408192	6.90E−05
ENSMUSG00000049130	*C5ar1*	2.449373	8.95E−21

ENSMUSG00000058715	*Fcer1g*	2.466747	4.70E−19
ENSMUSG00000079227	*Ccr5*	2.542427	2.95E−13
ENSMUSG00000031264	*Btk*	2.553092	3.00E−27
ENSMUSG00000025888	*Casp1*	2.562511	2.85E−19
ENSMUSG00000029915	*Clec5a*	2.563579	4.86E−05
ENSMUSG00000036526	*Card11*	2.701022	0.001801
ENSMUSG00000005087	*Cd44*	3.194564	6.31E−20
ENSMUSG00000003283	*Hck*	3.361242	5.31E−36
ENSMUSG00000004296	*Il12b*	3.362556	0.000641
ENSMUSG00000000982	*Ccl3*	3.541358	1.53E−16
ENSMUSG00000015854	*Cd5l*	3.569841	0.000767
ENSMUSG00000015437	*Gzmb*	3.580866	0.000633
ENSMUSG00000005413	*Hmox1*	3.656041	6.72E−08
ENSMUSG00000032076	*Cadm1*	3.906108	3.68E−18
ENSMUSG00000026981	*Il1rn*	4.900183	1.26E−14
ENSMUSG00000025473	*Adam8*	5.698634	7.67E−36

Supplementary Table 2. List of primers for qRT-PCR.

Gene Name	Primer Sequences
Inpp5d (Ship1)	Forward 5′-CAAGAGTCAGGAAGGAGAGAATG 3′
	Reverse 5′-GGGTCAGAGATAATG GGCTTTAG-3′
Tnf-α	Forward 5′-TGGGAGTAGATAAGGTACAGCCC-3′
	Reverse 5′-CATCTTCTCAAAACTCGAGTGACAA-3′
Mcp1	Forward 5′- CCACTCACCTGCTGCTACTCA-3′
	Reverse 5′- TGGTGATCCTCTTGTAGCTCTCC -3′
Arg1	Forward 5′-AAAGCTGGTCTGCTGGAAAA-3′
	Reverse 5′-ACAGACCGTGGGTTCTTCAC-3′
Il-10	Forward 5′- CCAGGGAGATCCTTTGATGA -3′
	Reverse 5′- AACTGGCCACAGTTTTCAGG -3′
Bax	Forward 5′-GAAGTTGCCATCAGCAAACAT-3′
	Reverse 5′-GGAGATGAACTGGACAGCAATA-3′
Bcl-2	Forward 5′-GAGACAGCCAGGAGAAATCAA-3′
	Reverse 5′-GTGGATGACTGAGTACCTGAAC-3′
CCR5	Forward 5′-CTTGTCATGGTCATCTGCTACTC-3′
	Reverse 5′-CCTGTGCCTCTTCTTCTCATTC-3′
CCL5	Forward 5′-GCCCACGTCAAGGAGTATTT-3′
	Reverse 5′-CTTGAACCCACTTCTTCTCTGG-3′

Supplementary Table 3. List of primary antibodies.

Antibody	Company	Catalog No.	Dilution	Applications	Source
**SHIP1**	Santa Cruz	sc-8425	1:1000, 1:200	WB<br>IHC, IF	Mouse
**Bax**	Santa Cruz	sc-7480	1:1,000	WB	Mouse
**Bcl-2**	Santa Cruz	sc-492	1:1000	WB	Rabbit
**Cleaved caspase-3**	Cell signaling	#9664	1:1,000	WB	Rabbit
**Perilipin 1**	Abcam	ab61682	1:200	IF	Goat
**CD11b**	Abcam	ab128797	1:200	IF	Rabbit
**CD68**	Santa Cruz	sc-20060	1:200	IHC	Mouse
**PI3K**	Santa Cruz	sc-1637	1:1000	WB	Mouse
**p-Akt**	Cell signaling	#9271	1:1000	WB	Rabbit
**t-Akt**	Santa Cruz	sc-8312	1:1000	WB	Rabbit
**p62**	SIGMA	P0067	1:1000	WB	Mouse
**LC3B**	SIGMA	L7543	1:1000	WB	Mouse
**LAMP1**	Santa Cruz	ab24170	1:1000<br>1:200	WB<br>IF	Rabbit
**α-tubulin**	Sigma-Aldrich	T5168	1:1000	WB	Mouse

IF, immunofluorescence; IHC, immunohistochemistry; WB, Western blot.

A

Biological Process (BP)

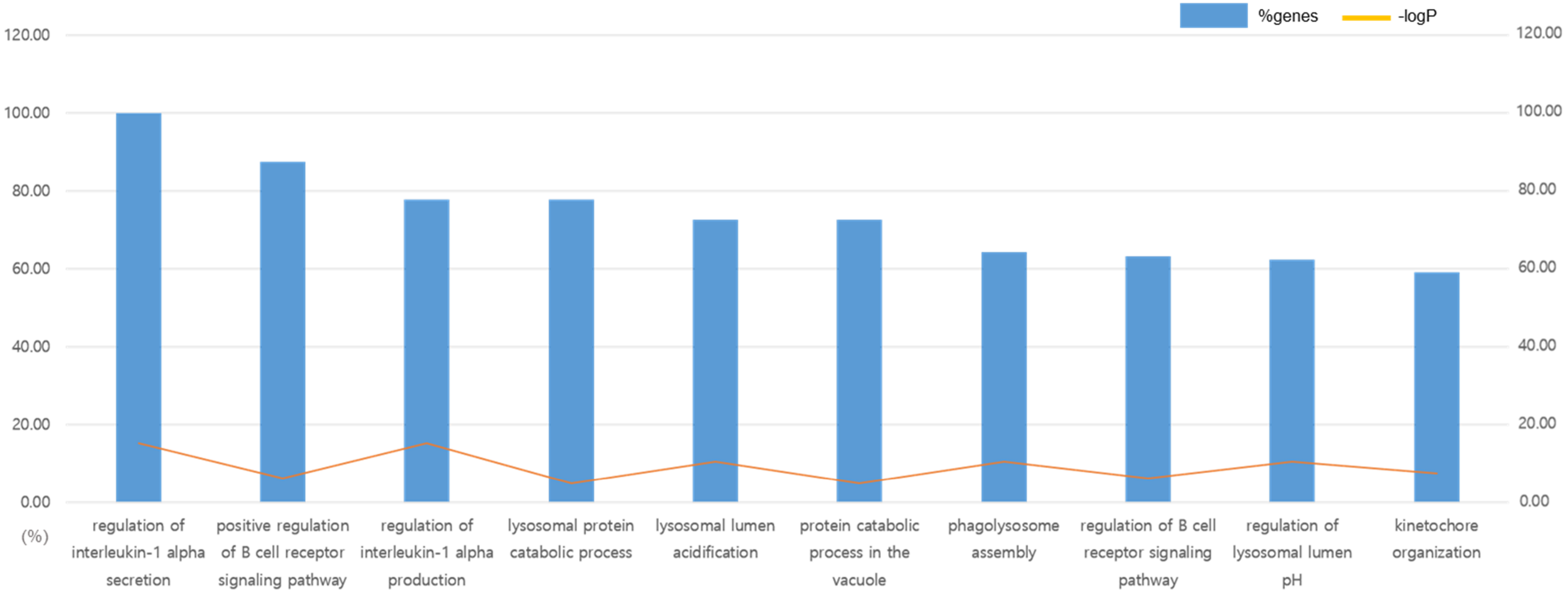

B

Cellular Components (CC)

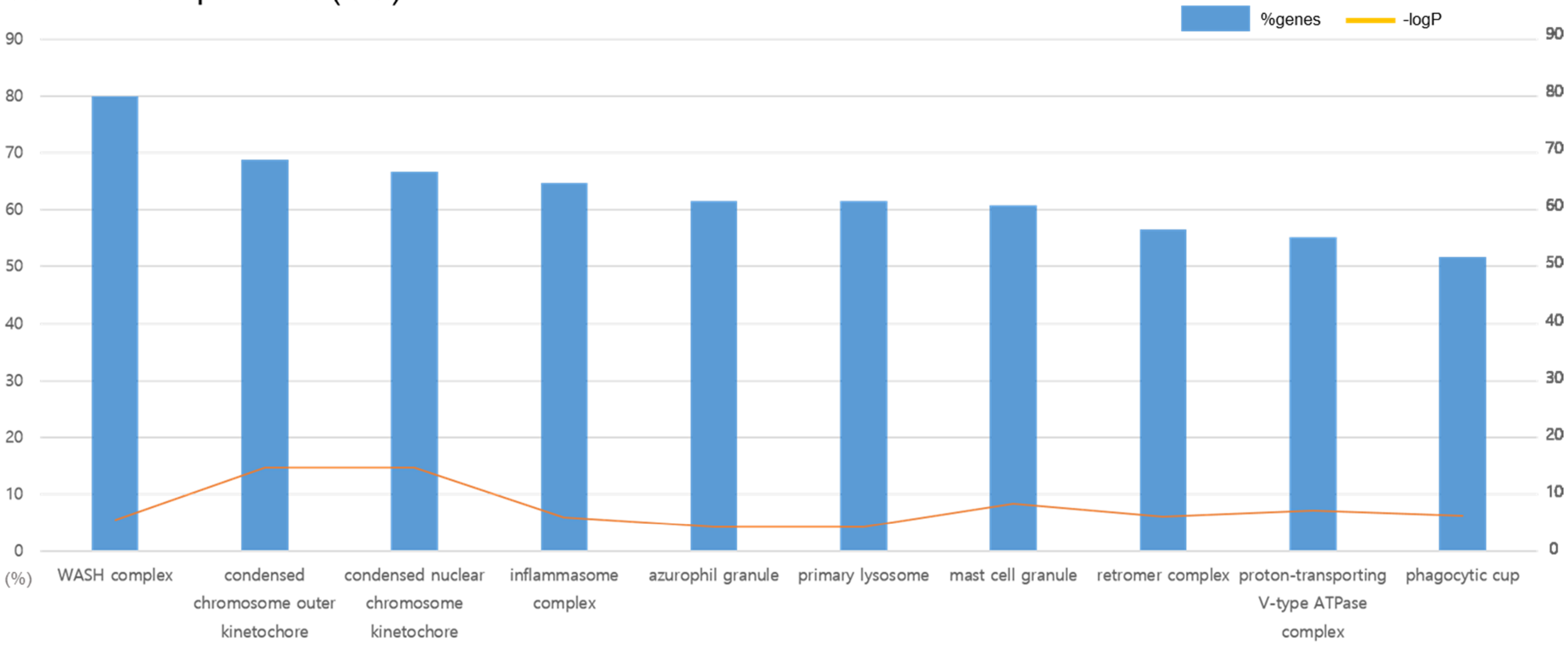

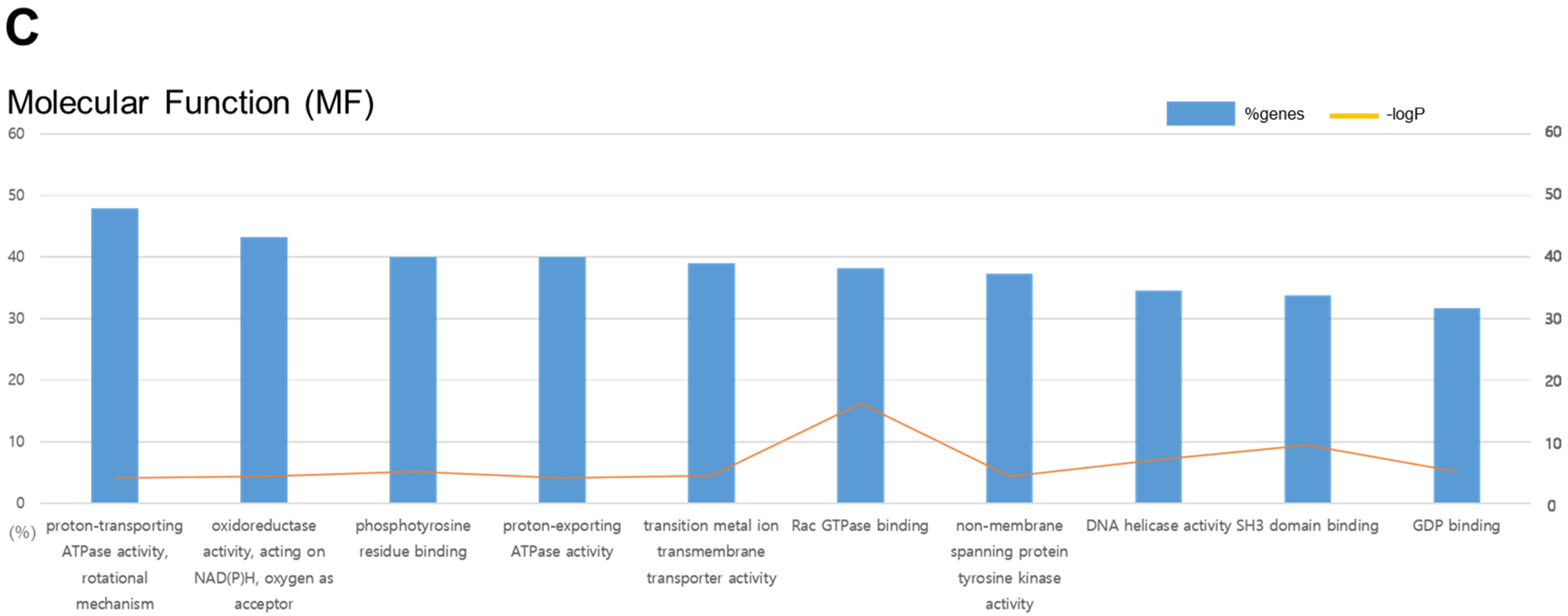

Supplementary Figure 1. Enrichment analysis of gene ontology (GO). Enriched GO terms of biological process (A), cellular component (B), and molecular function (C) using ClueGO pathway enrichment analysis in the adipose tissue of wild-type and obese (diet-induced or *ob*/*ob*) mice. Only representative GO terms that include more than two sibling terms are displayed. Blue bars indicate the proportion of associated genes, and yellow lines indicate −log (P value) of the enrichment analysis.

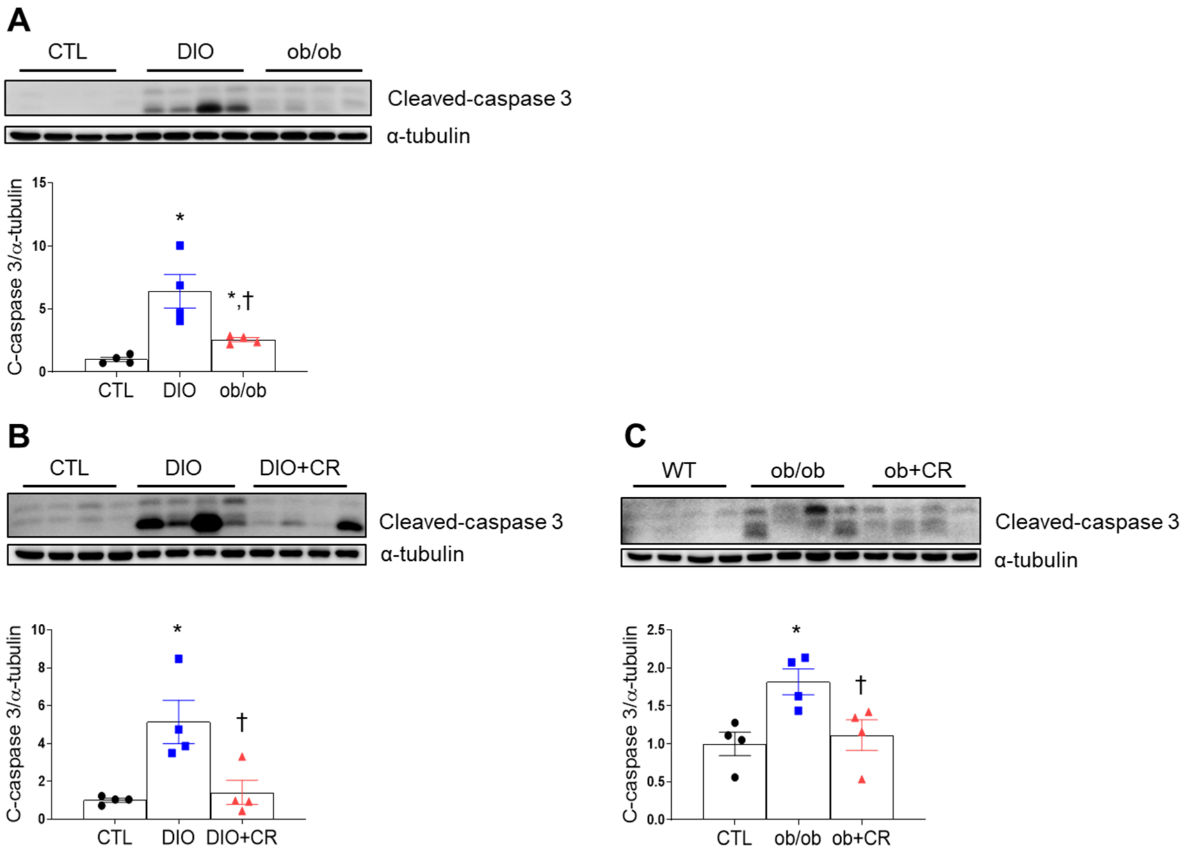

Supplementary Figure 2. Expression of cleaved caspase 3 in the adipose tissues of DIO and ob/ob mice. (A) Western blot and quantitative analysis of cleaved caspase 3 protein expression in CTL, DIO, and ob/ob mice. (B and C) Effect of caloric restriction on cleaved caspase 3 expression in the adipose tissues of DIO and ob/ob mice. Data are presented as the means ± SEM. *P < 0.05 versus control (CTL) or wild type (WT) mice; †P < 0.05 versus diet-induced obesity (DIO) or ob/ob mice. CTL (black circle), DIO (blue square), ob/ob (red triangle).
